# Development and evaluation of recombinase polymerase amplification combined with lateral flow dipstick assays for co-detection of epizootic haemorrhagic disease virus and the Palyam serogroup virus

**DOI:** 10.1186/s12917-021-02977-9

**Published:** 2021-08-25

**Authors:** Zhuo-ran Li, Zhen-xing Yang, Zhan-hong Li, Xiang Gao, Zhong-yan Hu, Heng Yang, De-fang Liao

**Affiliations:** 1 Yunnan Tropical and Subtropical Animal Virus Diseases Laboratory, Yunnan Animal Science and Veterinary Institute, Yunnan 650224 Kunming, China; 2 Animal Disease Control and Prevention Center of Jinghong, Yunnan 666100 Jinghong, China

**Keywords:** Epizootic haemorrhagic disease virus, The Palyam serogroup viruses, Recombinase polymerase amplification, Nucleic acid

## Abstract

**Background:**

Epizootic haemorrhagic disease virus (EHDV) and the Palyam serogroup viruses (PALV) have led to significant economic losses associated with livestock production globally. A rapid, sensitive and specific method for the detection of EHDV and PALV is critical for virus detection, monitoring, and successful control and elimination of related diseases.

**Results:**

In the present study, a recombinase polymerase amplification combined with lateral flow dipstick (RPA-LFD) assay for the co-detection of genome segment 1 (Seg-1) of EHDV and PALV was developed and evaluated. The analytical sensitivities of the established RPA-LFD assay in the detection of EHDV and PALV were 7.1 copies/µL and 6.8 copies/µL, respectively. No cross-reaction with other members of the genus *Orbivirus*, including African horse sickness virus, bluetongue virus, *Guangxi orbivirus, Tibet orbivirus* and *Yunnan orbivirus* was observed. The established RPA-LFD assay accurately detected 39 EHDV strains belonging to 5 serotypes and 29 PALV strains belonging to 3 serotypes. The trace back results of quantitative real-time polymerase chain reaction (qRT-PCR) and the established RPA-LFD assay on sentinel cattle were consistent. The coincidence rates of qRT-PCR and the established RPA-LFD assay in 56 blood samples from which EHDV or PALV had been isolated and 96 blood samples collected from cattle farms were more than 94.8 %. The results demonstrated that the established RPR-LFD assay is specific, sensitive and reliable, and could be applied in early clinical diagnosis of EHDV and PALV.

**Conclusions:**

This study highlights the development and application of the RPA-LFD assay in the co-detection of EHDV and PALV for the first time. The assay could be used as a potential optional rapid, reliable, sensitive and low-cost method for field diagnosis of EHDV and PALV.

**Supplementary Information:**

The online version contains supplementary material available at 10.1186/s12917-021-02977-9.

## Background

Epizootic haemorrhagic disease virus (EHDV) and the Palyam serogroup viruses (PALV) are members of the genus *Orbivirus* in the family *Reoviridae*, which exhibit some common morphological and structural characteristics [1, 2]. The genomes of the viruses consist of 10 double-stranded RNA segments (Seg-1–Seg-10) encoding seven structural (VP1–VP7) and four non-structural (NS1–NS3 and NS3a) proteins. The outer capsid proteins, VP2 and VP5, are responsible for viral serotypes [2, 3]. Unlike EHDV, which is transmitted by *Culicoides* midges, PALV is transmitted by a variety of arthropod vectors, such as mosquitoes, ticks and *Culicoides* midges [1, 2].

EHDV infection often leads to death in white-tailed deer and only Ibaraki virus belonging to EHDV-2 was previously known to cause bluetongue-like illness in cattle, whereas PALV is usually associated with abortion and teratogenesis in ruminants, principally cattle [2, 4, 5]. EHDV and PALV have contributed to considerable economic losses in livestock production sector globally; especially, EHDV-1,-6 and  -7, which have resulted in significant reductions in dairy production in Turkey, Israel and Japan over the last few years [6–10]. Several serotypes of EHDV (EHDV-1, -5, -6, -7 and -10) and PALV including Chuzan virus (CHUV), Bunyip Creek virus (BCV), and D’ Aguilar virus (DAV) are prevalent in China (unpublished data) [11–14]. In addition, EHDV and PALV can be transmitted through bites by blood-sucking midges of the *Culicoides* spp., thereby increasing risk of co-infection by the two viruses, which poses a potential threat to the cattle breeding industry in China.

Introduction of sensitive and specific diagnostic tests is critical for virus detection, monitoring, and effective control and elimination of orbiviral diseases. Accurate diagnosis presents a major challenge because the clinical symptoms associated with EHDV and PALV are generally non-specific or clinically inapparent [2, 15, 16]. Polymerase chain reaction (PCR) and enzyme-linked immunosorbent assay (ELISA) are the most routinely used techniques to detect pathogen nucleic acids and antibodies globally [17–19]. However, the techniques typically depend on expensive equipment and well-trained personnel, which in turn limits their current use in endemic field settings.

Over the last few decades, several isothermal amplification methods, such as loop-mediated isothermal amplification (LAMP) and recombinase polymerase amplification (RPA), have been developed and used to detect multiple pathogens [20–24]. Taking RPA as an example, an RPA reaction usually requires the participation of three major proteins, including a recombinase to separate DNA duplex, single-strand DNA-binding proteins to stabilize the open complex, and polymerase to synthesize DNA [24]. Although both LAMP and RPA are isothermal amplification methods, LAMP-mediated amplified reaction requires at least two pairs of primers, and its application in the co-detection of multiple pathogens is challenging due to formation of dimers between primers [25]. By contrast, one RPA reaction use only two opposing primers (with one labeled probe), and is achieved at a low and constant temperature. The amplified products can be detected by a specific lateral flow dipstick (LFD) and observed with naked eyes [23, 24]. LFD is a technology that utilizes antibodies to recognize the antigens incorporated into the amplified products, and presents results on the membrane carrier [22–24]. Therefore, RPA assay could present more potential prospects than other detection methods with reference to the detection efficiency and rapid on-site diagnosis.

In the present study, we aimed to develop an RPA-LFD assay for the co-detection of EHDV and PALV in clinical blood samples and to evaluate its efficacy in comparison with quantitative real-time polymerase chain reaction (qRT-PCR).

## Results

### Designing and screening of RPA primers and LFD-probe sets

**Fig. 1:**
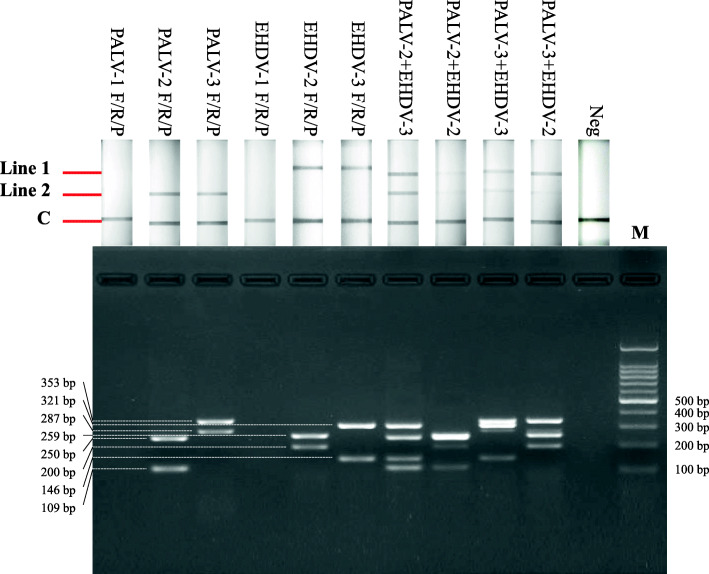
Screening of RPA-LFD primers and probe sets used for the co-detection of EHDV and PALV. Top: Results of RPA nfo reactions detected by LFD detectors; bottom: results of RPA nfo reactions analyzed by agarose gel electrophoresis. Lane 1–6: three sets of primers and probe used for the detection of EHDV and PALV, respectively; lane 7–10: four sets of primers and probes used for the co-detection of EHDV and PALV; lane 11: negative control; lane 12: 100 bp DNA marker (TIANGEN Biotech)

RPA primers and LFD-probes were designed against the highly conserved regions of Seg-1 and Seg-3 open reading frames of EHDV and PALV strains isolated from Asia and Australia (Table S1). To screen candidate primers and probes, EHDV-1 and CHUV genomic cDNAs served as templates, TwistAmp nfo reactions were performed at 39℃ for 20 min, and the amplified products subsequently analyzed using LFD detectors and 3 % agarose gel. Results revealed that primer sets of EHDV RPA-2, EHDV RPA-3, PALV RPA-2, and PALV RPA-3 with respective LFD-probe yielded specific amplifications for the established RPA-LFD assay, and generated products with expected size of 259 bp, 321 bp, 250 bp, and 353 bp (Fig. 1). RPA primers and LFD-probe sets of EHDV were paired with RPA primers and probe sets of PALV in succession, and subsequently evaluated the amplification and detection effects. EHDV RPA-3 primers and probe set in conjunction with PALV RPA-2 primers and probe set exhibited superior results (Fig. 1). RPA-LFD test lines of the primers and probe sets appeared in 10 min.

### Specificity and analytical sensitivity of the RPA-LFD assay

**Fig. 2:**
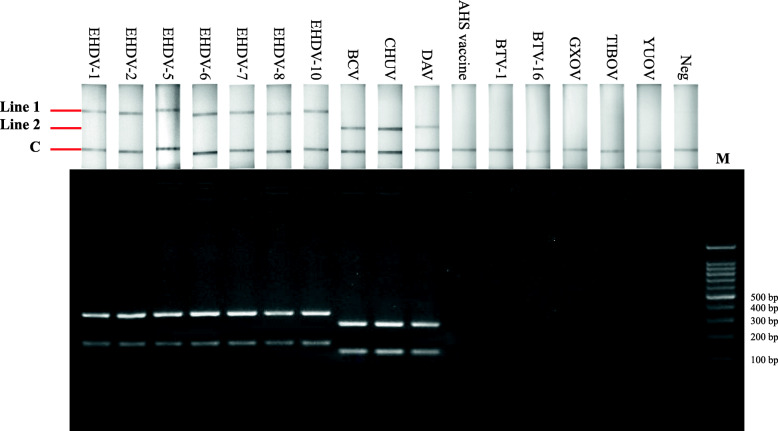
Specificity of the RPA-LFD assay in the detection of EHDV and PALV. Top: Results of RPA nfo reactions detected by LFD detectors; bottom: results of RPA nfo reaction analyzed by agarose gel electrophoresis. Lane 1–7: cDNAs of EHDV-1, -2, -5, -6, -7, -8 and − 10 strains served as templates; lane 8–10: cDNAs of BCV, CHUV and DAV strains served as templates; lane 11–16: cDNAs of AHS inactivated vaccine strain, BTV-1, -16 strains, GXOV, TIBOV and YUOV served as templates; lane 17: negative control; lane 18: 100 bp DNA marker

Considering that many different viruses are members of the *Orbivirus* genus and they share many similarities in genomic sequence characteristics, the specificity of the established RPA-LFD assay was determined by testing cDNAs transcribed from genomic RNAs of EHDV-1, -2, -5, -6, -7, -8, -10, BCV, CHUV, DAV, African horse sickness (AHS) inactivated vaccine, bluetongue virus serotype 1 (BTV-1) and BTV-16 strains, *Guangxi orbivirus* (GXOV), *Tibet orbivirus* (TIBOV) and *Yunnan orbivirus* (YUOV). Results revealed that none of the cDNAs of AHS inactivated vaccine, BTV-1, -16 strains, GXOV, TIBOV and YUOV exhibited positive results using the established RPA-LFD assay, which suggested that the primers and probe sets were specific for EHDV and PALV (Fig. 2).

**Fig. 3:**
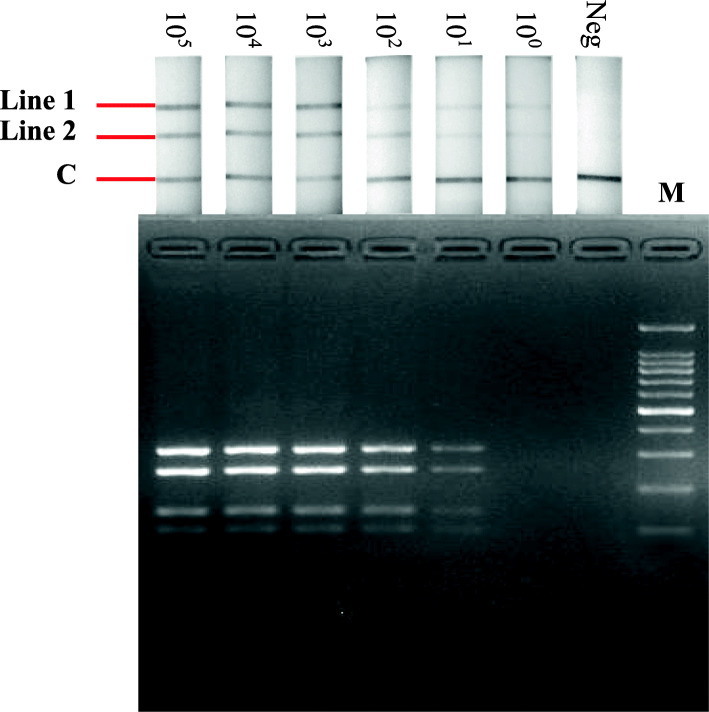
Analytical sensitivity of the RPA-LFD assay in the co-detection of EHDV and PALV. Top: Results of RPA nfo reactions detected by LFD detectors; bottom: results of RPA nfo reaction analyzed by agarose gel electrophoresis. Lane 1–6: a ten-fold serial dilution of purified EHDV and PALV Seg-1 DNA fragments from 10^5^ to 10^0^ copies/µL, which served as templates; lane 7: negative control; lane 8: 100 bp DNA marker

The concentrations of amplified and purified EHDV and PALV Seg-1 DNA fragments were 91.5 ng/µL and 71.3 ng/µL, respectively, and the quantities of copies were 7.1 × 10^13^ copies/µL and 6.8 × 10^13^ copies/µL, respectively. The analytical sensitivity of the established RPA-LFD assay was determined using a ten-fold serial dilution of purified EHDV and PALV Seg-1 DNA fragments from 10^0^ to 10^5^ copies/µL as templates, which were performed three times to ensure repeatability of the results. The results revealed that the established RPA-LFD assay could rapidly detect 7.1 copies of EHDV Seg-1 DNA and 6.8 copies of PALV Seg-1 DNA in 30 min (Fig. 3), which demonstrated the sensitivity and rapid performance of the RPA-LFD assay.

### Clinical performance of the RPA-LFD assay in the detection of EHDV and PALV

The established RPA assay could accurately detect a total of 68 strains of EHDV and PALV viruses (unpublished data), including 11 strains of EHDV-1, 9 strains of EHDV-5, 12 strains of EHDV-6, 4 strains of EHDV-7, 3 strains of EHDV-10, 7 strains of BCV, 17 strains of CHUV, and 5 strains of DAV (Table 1 and Table S2). The established RPA assay was subsequently used to test blood samples from which EHDV or PALV strains had been isolated between 2014 and 2019. qRT-PCR was initially employed to screen the samples because some blood samples have been stored at 4℃ for more than four years, and 56 samples with cycle threshold (CT) values below or equal to 38.0 were selected for the RPA-LFD assay. RPA assay failed to detect positive results in 2 of the 56 blood samples when compared to qRT-PCR, and the coincidence rate of the two detection assays was 96.4 % (Table 1 and Table S2).

**Table 1:** Reliability verification of RPA-LFD assay

			Serotypes of EHDV or PALV								Total
			**EHDV-1**	**EHDV-5**	**EHDV-6**	**EHDV-7**	**EHDV-10**	**BCV**	**CHUV**	**DAV**	
**Virus strains**	Number of isolated viruses		11	9	12	4	3	7	17	5	68
	RPA-LFD	Positive	11	9	12	4	3	7	17	5	68
		Negative	0	0	0	0	0	0	0	0	0
	Coincidence rate		39/39 × 100 %=100 %					29/29 × 100 %=100 %			68/68 × 100 %=100 %
**Blood samples**	CT values of qRT-PCR		28.9∼37.5	33.7∼37.8	29.5∼37.7	30.1∼38.0	32.6∼37.9	33.5∼37.6	28.2∼37.1	30.3∼36.3	
	RPA-LFD	Positive	8	7	9	4	3	6	12	5	54
		Negative	1	0	0	0	0	0	1	0	2
	Coincidence rate		31/32 × 100 %=96.9 %					23/24 × 100 %=95.8 %			54/56 × 100 %=96.4 %

**Fig. 4:**
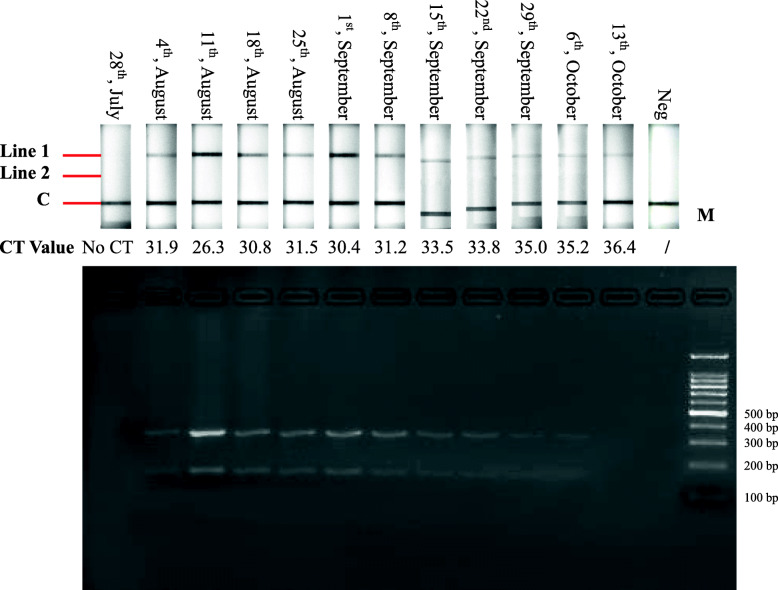
Trace back results of EHDV infection obtained from qRT-PCR and the RPA-LFD assay. Top: Results of RPA nfo reactions detected by LFD detectors; middle: results of qRT-PCR expressed with CT values; bottom: results of RPA nfo reactions analyzed by agarose gel electrophoresis. Lane 1–12: cDNAs of blood samples collected from sentinel animal infected with EHDV served as templates; lane 13: negative control; lane 14: 100 bp DNA marker

**Fig. 5:**
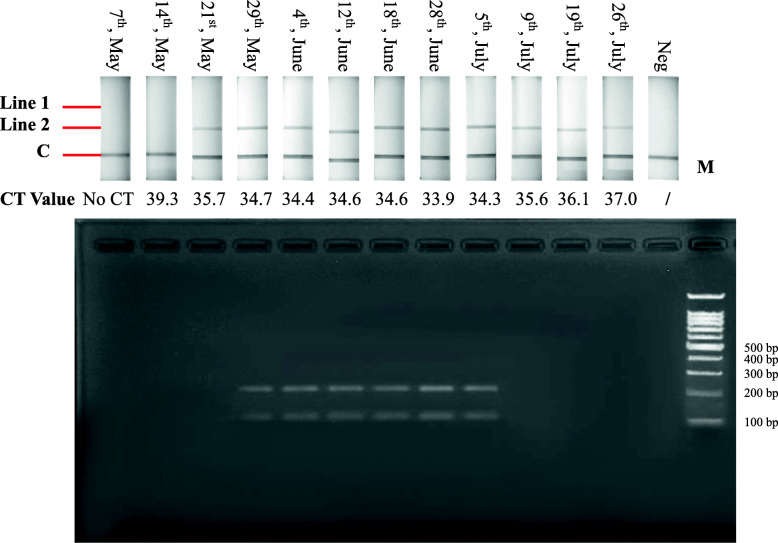
Trace back results of PALV infection obtained from qRT-PCR and the RPA-LFD assay. Top: Results of RPA nfo reactions detected by LFD detectors; Middle: results of qRT-PCR expressed with CT values; bottom: results of RPA nfo reactions analyzed by agarose gel electrophoresis. Lane 1–12: cDNAs of blood samples collected from sentinel animal infected with PALV served as templates; lane 13: negative control; lane 14: 100 bp DNA marker

The diagnostic validity of the established RPA-LFD assay was further evaluated by comparing test results of fresh clinical blood samples with those obtained using qRT-PCR. Total RNA was extracted from EDTA blood samples collected from sentinel cattle infected with EHDV or PALV in 2020, transcribed into cDNAs, and subsequently tested using the established RPA-LFD assay and qRT-PCR simultaneously. The RPA-LFD assay test results were basically consistent with those of qRT-PCR, except for the PALV detection results obtained on 14th, May 2020 (Figs. 4 and 5), in which qRT-PCR exhibited a CT value of 39.3, whereas the RPA-LFD assay detection results were negative (Fig. 5).

An additional 96 EDTA blood samples collected from cattle farms in Jinghong City were analyzed using qRT-PCR and the established RPA-LFD assay, respectively to determine the proportion of EHDV and PALV infection (Table S3). It was found that the infection rate of EHDV in cattle herds was 25.0 % (24/96), and the coincidence rate of qRT-PCR and the established RPA-LFD assay was 95.8 %. The infection rates of PALV in cattle herds obtained by qRT-PCR and the RPA-LFD assay were 20.8 % (20/96) and 19.8 % (19/96) respectively, and the coincidence rate was 96.9 %. The positive rates of EHDV and PALV co-infection detected by qRT-PCR and RPA-LFD assay were 13.5 % (13/96) and 12.5 % (12/96), respectively, and the coincidence rate was 94.8 % (Table 2 and Table S3).

**Table 2:** Infection rate of EHDV and PALV detected by qRT-PCR and RPA-LFD assay

		qRT-PCR					
		**EHDV**		**PALV**		**EHDV + PALV**	
		**Positive**	**Negative**	**Positive**	**Negative**	**Positive**	**Negative**
**RPA-LFD**	Positive	22	2	18	1	10	2
	Negative	2	70	2	75	3	81
**Coincidence rate**		(22 + 70)/96 × 100 %=95.8 %		(18 + 75)/96 × 100 %=96.9 %		(10 + 81)/96 × 100 %=94.8 %	

## Discussion

In the past, outbreaks of EHD and Chuzan virus-related diseases have caused considerable losses in the cattle industry in East Asia for many years. Presently, with the growth of global transportation networks and intensification of climate warming, the geographical range and active period of arthropod vectors have expanded, which could in turn lead to the spread of the arboviruses to higher-latitude regions, and in previously non-endemic areas. The monitoring system for the spread and prevalence of EHDV and PALV should be strengthened to prevent anticipated losses in the cattle industry [10–14, 26, 27].

Seg-1 and Seg-3 are relatively larger fragments in the 10 genome segments of the members of genus *Orbivirus*. The sequences of Seg-1 and Seg-3 are highly conserved within strains of identical *Orbivirus* species [2, 28, 29]. The characteristics of Seg-1 and Seg-3 ensure that appropriate primers and probes based on the specifications of the RPA method can be screened. Furthermore, a high degree of nucleotide sequence identity was observed among isolates of identical *Orbivirus* species from the same geographical region, and isolates of EHDV and PALV globally, can be segregated into distinct ‘eastern’ (Asia and Australia) and ‘western’ (Americas, Africa, Mediterranean Basin) topotypes [29, 30]. Consequently, primers and probes against the highly conserved regions of Seg-1 and Seg-3 belonging to the ‘eastern topotype’ were designed for the present study. The screening results of the primers and probe sets revealed that only the primers and probe sets targeting on Seg-1 obtained better co-detection effect, although the primers and probe sets targeting Seg-1 and Seg-3 performed well when EHDV or PALV were detected in isolation (Fig. 1).

The established RPA-LFD assay exhibited superior performance with reference to specificity and sensitivity experiments, and detected EHDV and PALV simultaneously to enhance detection efficiency (Figs. 2 and 3); moreover, the assay accurately detected in total of 68 virus strains including 5 serotypes of EHDV and 3 serotypes of PALV (Table 1); subsequently, the established assay was used to detect blood samples from which EHDV or PALV strains had been isolated and trace back infection dynamics in sentinel cattle. Detection results of 56 blood samples from which the viruses had been isolated revealed that the coincidence rates of the established RPA-LFD assay and qRT-PCR were 96.4 %, which implies that the RPA-LFD assay is reliable (Table 1). In trace back experiments, the detection results obtained using the established RPA-LFD assay were generally equivalent to those of qRT-PCR, except for a sample collected from sentinel animal infected with PALV on 14th, May 2020 (Figs. 4 and 5). We calculated the copy numbers of PALV in blood sample collected on 14th, May 2020 according to a regression equation of the PALV group-specific qRT-PCR [31], and established that the copy numbers were 0.39 per microliter, which suggested that the results obtained from qRT-PCR were negative. In summary, the RPA-LFD assay exhibited high sensitivity in the detection of clinical samples. In addition, the detection limits of LFD and agarose gel are 0.005 ng and 0.1 ng DNA, respectively, according to the manufacturer’s instructions on LFD detector and GoldView II, and samples with low viral nucleic acid contents could be detected by LFD after RPA amplification, although with no corresponding bands on agarose gel (Figs. 3, 4 and 5).

The sequence of RPA primers and LFD-probes used in the present study differed from those of the ‘western topotype’ strains; it was presumed that the primers and probes could not be used to detect ‘western topotype’ strains. However, due to the lack of corresponding nucleic acids, the ability of the RPA-LFD assay to detect ‘western topotype’ strains has not been evaluated. Specific primers and probes suitable for detecting ‘western topotype’ strains should be designed and validated using nucleic acids extracted from ‘western topotype’ strains to enhance the RPA-LFD assay. We initially reverse transcribed viral genomic RNAs, and subsequently performed RPA amplification and LFD detection because TwistDx does not provide a product that couples RNA reverse transcription and RPA reaction. If reverse transcription reactions could be combined with RPA nfo reactions, the detection system for viral genomic RNAs would be accomplished in one tube, which would in turn, enhance detection efficiency, prevent contamination and be more effective for field detection. Another product of TwistDx, TwistAmp Liquid Basic Kit, is known to be compatible with direct addition of reverse transcriptase, therefore we plan to combine reverse transcription and RPA amplification into one-tube by adding reverse transcriptase, which will avoid cross-contamination and further save time.

RPA-LFD has the advantages of low cost and high efficiency, but because RPA amplification is very sensitive, it is easy to cause cross-contamination due to aerosol during the operation. Furthermore, the RPA-LFD probe is generally more than 45 nucleotides in length, so it must be ensured that the target gene possesses sufficiently long conserved regions. For example, it was possible to design primers and TaqMan probe targeting Seg-9 of EHDV and obtain reliable detection results using qRT-PCR method [32], but the conserved regions of EHDV Seg-9 were too short to design RPA-LFD probes. In addition to the difficulty of probe design, the cost of synthesizing RPA-LFD probes is relatively higher than TaqMan probe. Therefore, we firstly determined the position of the probe through strict sequence alignment, and then selected the best primers and probe set by adjusting the positions of upstream and downstream primers. We plan to further evaluate the specificity and sensitivity of the established RPA-LFD method in the field to promote its application in on-site diagnosis.

The state of EHDV and PALV co-infection in cattle herds was only investigated in Jinghong City of Yunnan Province, and the co-infection rate was approximately 13 % reference to the detection results of qRT-PCR and RPA-LFD assay (Table 2). Therefore, further studies should be conducted to expand the scope of the present investigation to extensively understand the state of prevalence and co-infection of the two viruses in China.

## Conclusions

The present study developed an RPA-LFD assay for the co-detection of EHDV and PALV for the first time. Although further studies are required to evaluate the performance of RPA-LFD assay in field settings, the assay can be a potential alternative to conventional PCR method because it is simple, rapid, reliable, efficient, and low cost.

## Methods

### Viruses and blood samples

EHDV-1, -5, -6, -7, -10, BCV, CHUV, DAV, BTV, GXOV, TIBOV and YUOV strains were isolated from sentinel animal blood samples, *Culicodies* spp., or mosquitoes between 2012 and 2020, with support from the Special Fund for Agro-scientific Research in the Public Interest of China (unpublished data) [13, 14, 33–35]. International standard reference strains of EHDV-2, -8 and AHS inactivated vaccine strain were obtained from Elizabeth Macarthur Agricultural Institute, New South Wales, Australia.

EHDV, PALV and BTV strains were propagated in baby hamster kidney cells (BHK-21, China Center for Type Culture Collection, Wuhan, China), whereas GXOV, TIBOV and YUOV strains were propagated in *Aedes albopictus* cells (C6/36, China Center for Type Culture Collection). The supernatants of infected cells with 90 % CPE were clarified by centrifugation at 1,000 g for 10 min and stored at -80 °C.

Blood samples from which EHDV or PALV had been isolated between 2014 and 2019 were prepared for group-specific detection using qRT-PCR. Sentinel cattle free of arboviral nucleic acids and antibodies were set up in Menghan Town, Jinghong City, Yunnan Province, and blood sampled weekly between May and October 2020. Blood samples were transported to Yunnan Tropical and Subtropical Animal Virus Diseases Laboratory, Yunnan Province, China, for serological test, viral nucleic acid detection and virus isolation. Ninety-six EDTA blood samples were collected in Dapingzhang Cattle Raising Cooperative of Mengwang Village, Wumei Cattel Farm of Dadugang Village and Ganan Cattle Raising Cooperative of Menglong Town in Jinghong City, Yunnan Province (Table S3).

### RNA extraction and reverse transcription

Viral RNA was extracted from 200 µL infected cell culture supernatant using EasyPure Viral DNA/RNA Kit (TransGen, Beijing, China) according to the manufacturer’s instructions. RNA was extracted from 50 µL of blood samples using MagMAX magnetic beads viral RNA isolation kit on a KingFisher Flex platform (Applied Biosystems, Pittsburgh, PA, USA). The extracted RNA was denatured at 95℃ for 3 min and used as a template to synthesize cDNA through reverse transcription using PrimeScript™ RT Master Mix (Takara, Dalian, China) according to the manufacturer’s instructions. Viral cDNA was stored at -80 °C for further analyses.

### Designing of RPA primers and LFD-probes

Multiple sequence alignments of Asian and Australian EHDV and PALV strains available from the GenBank were performed to establish highly conserved regions of Seg-1 and Seg-3. RPA primers and LFD-probes were designed against the Seg-1 and Seg-3 consensus sequences of EHDV and PALV, respectively. RPA primers and LFD-probes were labeled according to the manufacturer’s instructions of TwistAmp™ nfo kit (TwistDx Limited), and synthesized by GENEray Biotechnology (Shanghai, China). The oligonucleotide backbone of LFD-probe included a 5’-antigenic labeled fluorescein isothiocyanate isomer (FITC) or digoxin (DIG) group, an internal abasic nucleotide analogue dSpacer (tetrahydrofuran, THF) and a 3’-polymerase extension blocking group C3-spacer. The lower primers were labeled with a 5’-antigen of biotin (Bio). Oligonucleotide sequences of RPA primers and LFD-probes are listed in Table S1.

### Screening of RPA primers and LFD-probes

cDNAs of EHDV-1 and CHUV were used as templates for screening RPA primers and LFD-probes. RPA reactions were performed using TwistAmp™ nfo kit. The freeze-dried enzyme pellet was dissolved in a solution containing 29.5 µL rehydration buffers, 2.1 µL of each primer (10 µM), 0.6 µL of each probe (10 µM), and 1 µL cDNA template. RNase-free water (Sangon Biotech, Shanghai, China) was added to the reaction system to adjust the volume to 47.5 µL, and 2.5 µL of magnesium acetate (280 mM) subsequently added. Assays were completed in a thermos metal bath (TIANGEN Biotech, Beijing, China) at 39℃ for 20 min in accordance with the TwistAmp™ nfo Kit quick guide. Amplified products were then analyzed using a 3 % (*w/v*) agarose gel electrophoresis to screen the optimal primers and probe sets. Agarose gel was supplemented with 1 × GoldView II (Solarbio, Beijing, China). The primers and respective probe for EHDV and PALV with specific amplification and detection effects were respectively paired. The co-detection ability of the established RPA-LFD assay was verified using the amplification system and analytical methods previously mentioned. RNase-free water served as a negative control.

PCRD Nucleic Acid Detector (Abingdon Health, York, UK), which is a sandwich immunochromatographic assay based on LFD, was used to visualize RPA amplification products. The detector contains three reaction lines, including a DIG/Bio labeled, a FITC/Bio labeled amplicon detection lines, and a flow-check control line. A total of 6 µL of the RPA products were diluted with 84 µL of PCRD extraction buffer, and subsequently transferred 75 µL of the diluted reaction mixture to the sample well of a PCRD test cassette. Positive EHDV or PALV nucleic acid results were indicated by the visualization of one detection line and a control line simultaneously perceptible on detectors in 10 min. Positive EHDV and PALV nucleic acid results were indicated by two detection lines and a control line, whereas the negative reactions only generated a control line.

### Generation of DNA molecular standards

Seg-1 DNA molecular standards containing RPA amplified regions of EHDV and PALV were amplified using primers listed in Table S1. The products were purified, sequenced, and used to determine the analytical sensitivity of the established RPA-LFD assay. Concentrations of DNA molecular standards were determined using NanoVue Plus (GE Healthcare, Chicago, IL, USA). The quantity of copies was calculated using the formula: DNA copy number (copies/µL) = (X/[a × 660]) × 6.022 × 10^23^, where X = g/µL of the DNA molecular standard concentration measured at a wavelength of 260 nm; a = DNA molecular standard length in nucleotides [32].

### Specificity and analytical sensitivity of the RPA-LFD assay

The specificity of the RPA-LFD assay was determined by testing other orbiviral pathogens, including genomic cDNAs of AHS inactivated vaccine strain, BTV-1, -16 strains, GXOV, TIBOV and YUOV. cDNAs of EHDV and PALV strains were used as positive controls. RNase-free water served as a negative control.

DNA molecular standards were serially diluted ten-fold ranging from 10^0^ to 10^5^ copies per microliter. The RPA reaction was performed and tested using agarose gel electrophoresis and PCRD detectors to determine DNA analytical sensitivity of established RPA-LFD assay. DNA molecular standard samples were analyzed using three independent assays. RNase-free water served as a negative control.

### Clinical performance of RPA-LFD assay in the detection of EHDV and PALV

cDNAs of 68 strains of EHDV and PALV isolated between 2014 and 2019 were used as templates to verify the reliability of the RPA-LFD assay (unpublished data, Table S2). Subsequently, blood samples from which EHDV or PALV had been isolated between 2014 and 2019 were initially screened by qRT-PCR, and the samples with CT values below or equal to 38.0 were analyzed using RPA-LFD assay to further verify the reliability of the established method. The CT values represented the average of three replicate wells.

qRT-PCR and the RPA-LFD assay were performed to trace back infection dynamics of the infected sentinel cattle to evaluate diagnostic ability of established RPA-LFD assay. RNA was extracted from EDTA blood samples collected in 2020 and reverse transcribed into cDNAs. Amplified products of RPA reaction were detected by agarose gel electrophoresis and LFD detectors. Primers and probes used for EHDV group-specific and PALV group specific qRT-PCR were synthesized as previously described by Li et al., [31] and Maan et al., [32].

Ninety-six EDTA blood samples collected from cattle farms in 2020 were analyzed using the established RPA-LFD assay and qRT-PCR to determine the ratio of EHDV and PALV co-infection. The qRT-PCR reactions performed using Luna Universal Probe qPCR Master Mix (NEB, Beijing, China) according to the manufacturer’s instructions. RNase-free water served as a negative control.

## Supplementary Information

**Additional file 1: Table S1. **Primers and probes used for RPA-LFD assay and amplification of DNA molecular standards.

**Additional file 2: Table S2.** qRT-PCR and RPA-LFD methods detection results of virus strains and blood samples.

**Additional file 3: Table S3.** qRT-PCR and RPA-LFD methods detection results of blood samples collected from farms.

## Data Availability

The datasets used and analysed during the current study are available from the corresponding author on reasonable request.

## Abbreviations

EHDV: Epizootic haemorrhagic disease virus
PALV: The Palyam serogroup viruses
RPA-LFD: Recombinase polymerase amplification combined with lateral flow dipstick
Seg-1: Genomic segment 1
qRT-PCR: Quantitative real-time polymerase chain reaction
ELISA: Enzyme-linked immunosorbent assay
CHUV: Chuzan virus
BCV: Bunyip Creek virus
DAV: D’ Aguilar virus
AHS: African horse sickness
BTV: bluetongue virus
GXOV: *Guangxi oribivirus*
TIBOV: *Tibet oribivirus*
YUOV: *Yunnan orbivirus*
CT: Cycle threshold
FITC: Fluorescein isothiocyanate isomer
DIG: Digoxin
THF: Tetrahydrofuran
Bio: Biotin
