# Supplementary material for: Development and evaluation of recombinase polymerase amplification combined with lateral flow dipstick assays for co-detection of epizootic haemorrhagic disease virus and the Palyam serogroup virus
Source: BMC Vet Res. 2021 Aug 25;17:286. doi: 10.1186/s12917-021-02977-9 (PMC8390197; doi:10.1186/s12917-021-02977-9)
Supplement: Supplementary file 1 — Additional file 1: Table S1. Primers and probes used for RPA-LFD assay and amplification of DNA molecular standards. [file 12917_2021_2977_MOESM1_ESM.docx]

**Table S1** Primers and probes used for RPA-LFD assay and amplification of DNA molecular standards

| **Primer and**  **probe names** | **Target genes** | **Primer and probe sequences (5’-3’) ^a^** | **Nucleotide location** | **PCR products size (bp)** |
| --- | --- | --- | --- | --- |
| EHDV RPA-1 F | Seg-3 | AGCAAGGTTACAAGCGAATATGGGTCAATTACGGG | 2238-2272 | 216 |
| EHDV RPA-1 R |  | Bio-TCGTTAGCATATTTAATCGTAGCATAGGTTAGCCC | 2419-2453 |  |
| EHDV LFD-1 Probe |  | DIG-GGGCGGCTTTACGAAGAATTATGGATGACG[THF]AGGTTGGGTTAGGTT[C3-spacer] | 2270-2315 | 184 |
| EHDV RPA-2 F | Seg-1 | TTGATAGTTACATTGCCATTAAACGAATATTTTGC | 1597-1631 | 259 |
| EHDV RPA-2 R |  | Bio-TTGCTTCACGAATACCTTCTAACATACCCCTCCGA | 1821-1855 |  |
| EHDV LFD-2 Probe |  | DIG-CGAGTATAAGAAATTGGGCGGTAAAATAAT[THF]GTAGGAGATCTCGAG[C3-spacer] | 1656-1701 | 200 |
| EHDV RPA-3 F | Seg-1 | TGGATTGCCTGTTTTACATGATTCAACTTGGGA | 213-245 | 321 |
| EHDV RPA-3 R |  | Bio-TAACGCTTGACGAACGATGACATAACTTTCACCCCTAAAC | 494-533 |  |
| EHDV LFD-3 Probe |  | DIG-TTTATTGAACAAAGAGCGAAGAACGAAATG[THF]AAATATATGGAGACA[C3-spacer] | 388-433 | 146 |
| PALV RPA-1 F | Seg-1 | CGGATAATCAGATTTTAGTATCGCCTGTAGATGGA | 2089-2123 | 495 |
| PALV RPA-1 R |  | Bio-ATCAACATCATTCTATCCTGGGGTATATAT | 2554-2583 |  |
| PALV LFD-1 Probe |  | FITC-TACAAGAGGAGGTGAAACATCGGTGCGCTG[THF]GTTAATCTCATTTCT[C3-spacer] | 2216-2261 | 368 |
| PALV RPA-2 F | Seg-1 | TACTAAAATTTCAAGAGGGTTTTCTCATAAATTGG | 2553-2587 | 250 |
| PALV RPA-2 R |  | Bio-GGTCATAACTATATTAAGCGATTCATAATAAGCCCC | 2767-2802 |  |
| PALV LFD-2 Probe |  | FITC-TGAGGATGGTTTTACTATGTATTTAATTCG[THF]GATCCATTGTGTGCT[C3-spacer] | 2694-2739 | 109 |
| PALV RPA-3 F | Seg-3 | GTAATACAGAGAGCAATACAGTGGTTAGTGAGACT | 697-731 | 353 |
| PALV RPA-3 R |  | Bio-ATTAATGCTAAATATATCTTTTTAACATCATC | 1018-1049 |  |
| PALV LFD-3 Probe |  | FITC-GATTTTATGACTGATTTTCGGAGGGCGGAT[THF]CTATATGGATACTTAC[C3-spacer] | 763-809 | 287 |
| EHDV RPA Sen F | Seg-1 | AAAATGCAATGGTCGCAATTACCGT | −8-17 | 1174 |
| EHDV RPA Sen R |  | TTTTTCACCCACGCACGTCC | 1147-1166 |  |
| PALV RPA Sen F | Seg-1 | GTCATATTGCTTCTGCTTCAA | 2375-2395 | 959 |
| PALV RPA Sen R |  | CCTTACCCGTGTGCTCATCC | 3314-3333 |  |

^a^ Primer and probe modifications: *Bio* Biotin, *DIG* Digoxin, *FITC* Fluorescein isothiocyanate isomer, *THF* Tetrahydrofuran, *C3-spacer* 3’ phosphate blocker
