# Supplementary material for: Development and evaluation of recombinase polymerase amplification combined with lateral flow dipstick assays for co-detection of epizootic haemorrhagic disease virus and the Palyam serogroup virus
Source: BMC Vet Res. 2021 Aug 25;17:286. doi: 10.1186/s12917-021-02977-9 (PMC8390197; doi:10.1186/s12917-021-02977-9)
Supplement: Supplementary file 2 — Additional file 2: Table S2. qRT-PCR and RPA-LFD methods detection results of virus strains and blood samples. [file 12917_2021_2977_MOESM2_ESM.docx]

**Table S2** qRT-PCR and RPA-LFD methods detection results of virus strains and blood samples

| **Serotypes** | **Virus strains** | **Collection date** | **Collection locations** | **CT values** | | **RPA-LFD results** | |
| --- | --- | --- | --- | --- | --- | --- | --- |
|  |  |  |  | **Virus strains** | **Blood samples** | **Virus strains** | **Blood samples** |
| BCV | V145 | 2014 | Qujing, Yunnan Province | 21.7 | 38.5 | positive | NA |
| BCV | V104 | 2014 | Qujing, Yunnan Province | 21.6 | 37.6 | positive | positive |
| BCV | V256 | 2015 | Shantou, Guangdong Province | 20.3 | 36.3 | positive | positive |
| BCV | V252 | 2016 | Shantou, Guangdong Province | 21.9 | 34.6 | positive | positive |
| BCV | V253 | 2016 | Shantou, Guangdong Province | 22.8 | 34.0 | positive | positive |
| BCV | V254 | 2016 | Shantou, Guangdong Province | 23.1 | 33.8 | positive | positive |
| BCV | V255 | 2016 | Shantou, Guangdong Province | 22.4 | 33.5 | positive | positive |
| CHUV | V143 | 2014 | Qujing, Yunnan Province | 21.1 | No CT | positive | NA |
| CHUV | V144 | 2014 | Qujing, Yunnan Province | 20.9 | 38.2 | positive | NA |
| CHUV | V103 | 2014 | Qujing, Yunnan Province | 21.7 | 39.1 | positive | NA |
| CHUV | V078 | 2014 | Qujing, Yunnan Province | 18.4 | 38.6 | positive | NA |
| CHUV | V099 | 2014 | Qujing, Yunnan Province | 20.5 | 37.1 | positive | positive |
| CHUV | V100 | 2014 | Qujing, Yunnan Province | 22.1 | 36.8 | positive | negative |
| CHUV | V102 | 2014 | Qujing, Yunnan Province | 20.2 | 36.1 | positive | positive |
| CHUV | V101 | 2014 | Qujing, Yunnan Province | 19.7 | 36.5 | positive | positive |
| CHUV | V105 | 2014 | Qujing, Yunnan Province | 21.9 | 36.3 | positive | positive |
| CHUV | V141 | 2014 | Qujing, Yunnan Province | 21.2 | 35.6 | positive | positive |
| CHUV | V142 | 2014 | Qujing, Yunnan Province | 21.6 | 35.9 | positive | positive |
| CHUV | V098 | 2014 | Qujing, Yunnan Province | 22.0 | 35.7 | positive | positive |
| CHUV | V156 | 2015 | Honghe Hani and Yi Autonomous Prefecture, Yunnan Province | 21.6 | 34.4 | positive | positive |
| CHUV | V268 | 2017 | Qujing, Yunnan Province | 22.2 | 32.5 | positive | positive |
| CHUV | V300 | 2019 | Xishuangbanna Dai Autonomous Prefecture, Yunnan Province | 20.5 | 28.7 | positive | positive |
| CHUV | V304 | 2019 | Xishuangbanna Dai Autonomous Prefecture, Yunnan Province | 22.8 | 28.6 | positive | positive |
| CHUV | V302 | 2019 | Xishuangbanna Dai Autonomous Prefecture, Yunnan Province | 21.8 | 28.2 | positive | positive |
| DAV | V146 | 2014 | Qujing, Yunnan Province | 22.3 | 36.3 | positive | positive |
| DAV | V106 | 2014 | Qujing, Yunnan Province | 22.5 | 36.0 | positive | positive |
| DAV | V157 | 2015 | Honghe Hani and Yi Autonomous Prefecture, Yunnan Province | 22.0 | 35.1 | positive | positive |
| DAV | V203 | 2015 | Qujing, Yunnan Province | 21.9 | 34.4 | positive | positive |
| DAV | V284 | 2019 | Xishuangbanna Dai Autonomous Prefecture, Yunnan Province | 23.1 | 30.3 | positive | positive |
| EHDV-1 | V128 | 2014 | Shantou, Guangdong Province | 20.0 | 38.5 | positive | NA |
| EHDV-1 | V083 | 2014 | Qujing, Yunnan Province | 17.8 | No CT | positive | NA |
| EHDV-1 | V132 | 2014 | Dehong Dai and Jingpo Autonomous Prefecture, Yunnan Province | 16.3 | 37.5 | positive | negative |
| EHDV-1 | V194 | 2016 | Beihai, Guangxi Zhuang Autonomous Region | 19.2 | 34.0 | positive | positive |
| EHDV-1 | V195 | 2016 | Beihai, Guangxi Zhuang Autonomous Region | 21.8 | 33.8 | positive | positive |
| EHDV-1 | V281 | 2016 | Qujing, Yunnan Province | 20.9 | 33.4 | positive | positive |
| EHDV-1 | V294 | 2019 | Qujing, Yunnan Province | 22.1 | 29.7 | positive | positive |
| EHDV-1 | V295 | 2019 | Qujing, Yunnan Province | 20.3 | 29.1 | positive | positive |
| EHDV-1 | V297 | 2019 | Xishuangbanna Dai Autonomous Prefecture, Yunnan Province | 22.5 | 29.1 | positive | positive |
| EHDV-1 | V298 | 2019 | Xishuangbanna Dai Autonomous Prefecture, Yunnan Province | 21.6 | 29.0 | positive | positive |
| EHDV-1 | V303 | 2019 | Xishuangbanna Dai Autonomous Prefecture, Yunnan Province | 22.4 | 28.9 | positive | positive |
| EHDV-5 | V125 | 2014 | Nanning, Guangxi Zhuang Autonomous Region | 22.5 | No CT | positive | NA |
| EHDV-5 | V126 | 2014 | Nanning, Guangxi Zhuang Autonomous Region | 23.1 | No CT | positive | NA |
| EHDV-5 | V129 | 2014 | Nanning, Guangxi Zhuang Autonomous Region | 22.8 | 37.8 | positive | positive |
| EHDV-5 | V023 | 2014 | Dehong Dai and Jingpo Autonomous Prefecture, Yunnan Province | 22.5 | 37.5 | positive | positive |
| EHDV-5 | V024 | 2014 | Dehong Dai and Jingpo Autonomous Prefecture, Yunnan Province | 22.7 | 36.4 | positive | positive |
| EHDV-5 | V271 | 2014 | Puer, Yunnan Province | 22.1 | 35.7 | positive | positive |
| EHDV-5 | V270 | 2014 | Puer, Yunnan Province | 22.3 | 35.2 | positive | positive |
| EHDV-5 | V127 | 2015 | Liuzhou, Guangxi Zhuang Autonomous Region | 21.0 | 35.6 | positive | positive |
| EHDV-5 | V275 | 2016 | Qujing, Yunnan Province | 20.2 | 33.7 | positive | positive |
| EHDV-6 | V003 | 2014 | Qujing, Yunnan Province | 22.5 | 37.7 | positive | positive |
| EHDV-6 | V274 | 2014 | Puer, Yunnan Province | 20.6 | 39.0 | positive | NA |
| EHDV-6 | V273 | 2015 | Qujing, Yunnan Province | 22.3 | 38.7 | positive | NA |
| EHDV-6 | V276 | 2015 | Qujing, Yunnan Province | 21.5 | 38.1 | positive | NA |
| EHDV-6 | V258 | 2016 | Shantou, Guangdong Province | 22.4 | 34.2 | positive | positive |
| EHDV-6 | V260 | 2016 | Shantou, Guangdong Province | 21.8 | 33.8 | positive | positive |
| EHDV-6 | V261 | 2016 | Shantou, Guangdong Province | 22.7 | 33.5 | positive | positive |
| EHDV-6 | V259 | 2016 | Shantou, Guangdong Province | 22.1 | 33.1 | positive | positive |
| EHDV-6 | V262 | 2016 | Shantou, Guangdong Province | 22.5 | 33.6 | positive | positive |
| EHDV-6 | V263 | 2016 | Shantou, Guangdong Province | 21.7 | 33.7 | positive | positive |
| EHDV-6 | V283 | 2016 | Qujing, Yunnan Province | 23.0 | 32.8 | positive | positive |
| EHDV-6 | V285 | 2019 | Xishuangbanna Dai Autonomous Prefecture, Yunnan Province | 20.9 | 29.5 | positive | positive |
| EHDV-7 | V269 | 2014 | Qujing, Yunnan Province | 21.3 | 38.0 | positive | positive |
| EHDV-7 | V278 | 2015 | Puer, Yunnan Province | 22.4 | 35.6 | positive | positive |
| EHDV-7 | V286 | 2019 | Xishuangbanna Dai Autonomous Prefecture, Yunnan Province | 21.8 | 30.7 | positive | positive |
| EHDV-7 | V306 | 2019 | Qujing, Yunnan Province | 22.6 | 30.1 | positive | positive |
| EHDV-10 | V277 | 2014 | Qujing, Yunnan Province | 22.9 | 37.9 | positive | positive |
| EHDV-10 | V282 | 2016 | Qujing, Yunnan Province | 22.1 | 33.7 | positive | positive |
| EHDV-10 | V280 | 2017 | Qujing, Yunnan Province | 23.4 | 32.6 | positive | positive |

*NA* indicates that the established RPA-LFD method was not used to detect the blood samples.
