# Supplementary material for: Development and evaluation of recombinase polymerase amplification combined with lateral flow dipstick assays for co-detection of epizootic haemorrhagic disease virus and the Palyam serogroup virus
Source: BMC Vet Res. 2021 Aug 25;17:286. doi: 10.1186/s12917-021-02977-9 (PMC8390197; doi:10.1186/s12917-021-02977-9)
Supplement: Supplementary file 3 — Additional file 3: Table S3. qRT-PCR and RPA-LFD methods detection results of blood samples collected from farms. [file 12917_2021_2977_MOESM3_ESM.docx]

**Table S3** qRT-PCR and RPA-LFD methods detection results of blood samples collected from farms

| **Sample ID** | **Collection locations** | **CT values** | | **RPA results** | |
| --- | --- | --- | --- | --- | --- |
|  |  | **EHDV** | **PALV** | **EHDV** | **PALV** |
| 01 | Mengwang Village | No CT | No CT | negative | negative |
| 02 | Mengwang Village | No CT | No CT | negative | negative |
| 03 | Mengwang Village | 37.7 | 37.0 | negative | positive |
| 04 | Mengwang Village | No CT | No CT | negative | negative |
| 05 | Mengwang Village | 37.1 | 32.9 | positive | positive |
| 06 | Mengwang Village | No CT | No CT | negative | negative |
| 07 | Mengwang Village | No CT | No CT | negative | negative |
| 08 | Mengwang Village | 34.2 | No CT | positive | negative |
| 09 | Mengwang Village | No CT | No CT | negative | negative |
| 10 | Mengwang Village | No CT | No CT | negative | negative |
| 11 | Mengwang Village | No CT | No CT | negative | negative |
| 12 | Mengwang Village | 36.2 | No CT | positive | negative |
| 13 | Mengwang Village | No CT | No CT | negative | negative |
| 14 | Mengwang Village | 36.6 | No CT | positive | negative |
| 15 | Mengwang Village | No CT | No CT | negative | negative |
| 16 | Mengwang Village | No CT | No CT | negative | negative |
| 17 | Mengwang Village | No CT | 31.9 | negative | positive |
| 18 | Mengwang Village | No CT | No CT | negative | negative |
| 19 | Mengwang Village | 30.4 | 37.8 | positive | positive |
| 20 | Mengwang Village | No CT | No CT | negative | negative |
| 21 | Mengwang Village | No CT | No CT | negative | negative |
| 22 | Mengwang Village | No CT | No CT | negative | negative |
| 23 | Mengwang Village | No CT | No CT | negative | negative |
| 24 | Mengwang Village | No CT | No CT | negative | negative |
| 25 | Dadugang Village | No CT | No CT | negative | negative |
| 26 | Dadugang Village | 36.7 | 35.0 | positive | positive |
| 27 | Dadugang Village | No CT | No CT | negative | negative |
| 28 | Dadugang Village | No CT | No CT | negative | negative |
| 29 | Dadugang Village | No CT | No CT | negative | negative |
| 30 | Dadugang Village | No CT | No CT | negative | negative |
| 31 | Dadugang Village | No CT | No CT | negative | negative |
| 32 | Dadugang Village | 37.7 | 32.5 | positive | positive |
| 33 | Dadugang Village | No CT | No CT | negative | negative |
| 34 | Dadugang Village | No CT | No CT | negative | negative |
| 35 | Dadugang Village | 37.0 | No CT | positive | negative |
| 36 | Dadugang Village | No CT | No CT | negative | negative |
| 37 | Dadugang Village | No CT | No CT | negative | negative |
| 38 | Dadugang Village | 34.8 | No CT | positive | negative |
| 39 | Dadugang Village | No CT | 33.0 | positive | positive |
| 40 | Dadugang Village | No CT | 35.9 | negative | positive |
| 41 | Dadugang Village | No CT | No CT | negative | negative |
| 42 | Dadugang Village | No CT | No CT | negative | negative |
| 43 | Dadugang Village | No CT | No CT | negative | negative |
| 44 | Dadugang Village | 32.4 | 37.5 | positive | positive |
| 45 | Dadugang Village | No CT | No CT | negative | negative |
| 46 | Dadugang Village | No CT | No CT | negative | negative |
| 47 | Dadugang Village | No CT | No CT | negative | negative |
| 48 | Dadugang Village | 35.1 | No CT | positive | negative |
| 49 | Dadugang Village | No CT | No CT | negative | negative |
| 50 | Dadugang Village | No CT | No CT | negative | negative |
| 51 | Dadugang Village | No CT | No CT | negative | positive |
| 52 | Dadugang Village | 36.2 | 37.5 | positive | negative |
| 53 | Dadugang Village | No CT | No CT | negative | negative |
| 54 | Dadugang Village | No CT | No CT | negative | negative |
| 55 | Dadugang Village | No CT | No CT | negative | negative |
| 56 | Dadugang Village | No CT | No CT | negative | negative |
| 57 | Dadugang Village | No CT | No CT | negative | negative |
| 58 | Menglong Town | No CT | No CT | negative | negative |
| 59 | Menglong Town | No CT | No CT | negative | negative |
| 60 | Menglong Town | 37.5 | 32.1 | positive | positive |
| 61 | Menglong Town | No CT | No CT | negative | negative |
| 62 | Menglong Town | No CT | No CT | negative | negative |
| 63 | Menglong Town | No CT | No CT | negative | negative |
| 64 | Menglong Town | 35.8 | No CT | positive | negative |
| 65 | Menglong Town | No CT | No CT | negative | negative |
| 66 | Menglong Town | No CT | No CT | negative | negative |
| 67 | Menglong Town | No CT | No CT | negative | negative |
| 68 | Menglong Town | No CT | No CT | negative | negative |
| 69 | Menglong Town | No CT | No CT | negative | negative |
| 70 | Menglong Town | 36.9 | 31.8 | positive | positive |
| 71 | Menglong Town | No CT | 37.9 | negative | negative |
| 72 | Menglong Town | 35.9 | No CT | positive | negative |
| 73 | Menglong Town | No CT | No CT | negative | negative |
| 74 | Menglong Town | No CT | 35.7 | negative | positive |
| 75 | Menglong Town | No CT | No CT | negative | negative |
| 76 | Menglong Town | No CT | 33.2 | positive | positive |
| 77 | Menglong Town | No CT | No CT | negative | negative |
| 78 | Menglong Town | No CT | No CT | negative | negative |
| 79 | Menglong Town | 32.7 | 37.9 | positive | positive |
| 80 | Menglong Town | 34.9 | No CT | positive | negative |
| 81 | Menglong Town | No CT | No CT | negative | negative |
| 82 | Menglong Town | No CT | No CT | negative | negative |
| 83 | Menglong Town | No CT | No CT | negative | negative |
| 84 | Menglong Town | 36.0 | No CT | positive | negative |
| 85 | Menglong Town | No CT | No CT | negative | negative |
| 86 | Menglong Town | No CT | No CT | negative | negative |
| 87 | Menglong Town | 37.8 | 36.3 | negative | positive |
| 88 | Menglong Town | No CT | No CT | negative | negative |
| 89 | Menglong Town | No CT | 37.6 | negative | positive |
| 90 | Menglong Town | 36.7 | 33.3 | positive | positive |
| 91 | Menglong Town | No CT | No CT | negative | negative |
| 92 | Menglong Town | No CT | No CT | negative | negative |
| 93 | Menglong Town | 33.8 | No CT | positive | negative |
| 94 | Menglong Town | No CT | No CT | negative | negative |
| 95 | Menglong Town | No CT | No CT | negative | negative |
| 96 | Menglong Town | 33.5 | 37.6 | positive | positive |
